# Voltage-gated calcium channel α _2_δ subunits: an assessment of proposed novel roles

**DOI:** 10.12688/f1000research.16104.1

**Published:** 2018-11-21

**Authors:** Annette C. Dolphin

**Affiliations:** 1 Department of Neuroscience, Physiology and Pharmacology, University College London, Gower Street, London, WC1E 6BT, UK

**Keywords:** calcium channel, alpha2delta, interaction

## Abstract

Voltage-gated calcium (Ca_V_) channels are associated with β and α_2_δ auxiliary subunits. This review will concentrate on the function of the α_2_δ protein family, which has four members. The canonical role for α_2_δ subunits is to convey a variety of properties on the Ca_V_1 and Ca_V_2 channels, increasing the density of these channels in the plasma membrane and also enhancing their function. More recently, a diverse spectrum of non-canonical interactions for α_2_δ proteins has been proposed, some of which involve competition with calcium channels for α_2_δ or increase α_2_δ trafficking and others which mediate roles completely unrelated to their calcium channel function. The novel roles for α_2_δ proteins which will be discussed here include association with low-density lipoprotein receptor-related protein 1 (LRP1), thrombospondins, α-neurexins, prion proteins, large conductance (big) potassium (BK) channels, and *N*-methyl-d-aspartate (NMDA) receptors.

##  Introduction

**Figure 1.**
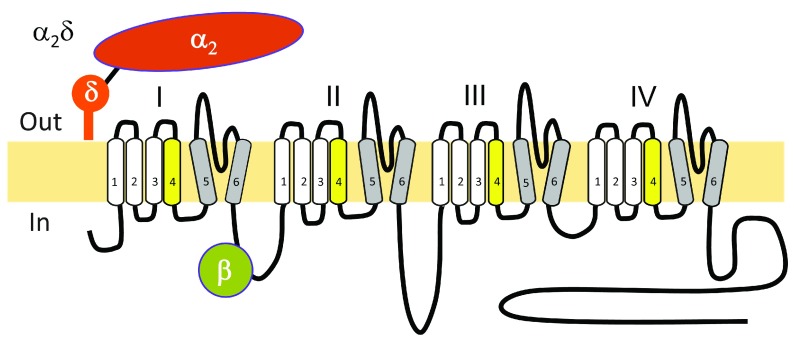
The subunit structure of voltage-gated calcium channels of the Ca _V_1 and Ca _V_2 family. The Ca _V_ α1 subunit with 24 transmembrane segments and the intracellular β and the extracellular α _2_δ subunits are shown. The γ subunit (γ1) is associated with Ca _V_1.1 only and is not depicted.

Voltage-gated calcium (Ca _V_) channels are ubiquitously present in excitable cells and are essential for their function. They can be divided into three classes (Ca _V_1–3). All except the Ca _V_3 (T type) channels are associated with several auxiliary subunits—termed α _2_δ and β—together with an additional γ subunit in skeletal muscle ^1, 2^ ( Figure 1). One of these subunits, α _2_δ, conveys a variety of properties on the channels but recently has also been reported to have distinct effects on both other ion channels and other biological processes. These novel aspects of α _2_δ function are the subject of this review. This topic is important, as α _2_δ-1 is the therapeutic target of the α _2_δ ligand (gabapentinoid) class of drugs ^3, 4^, which are widely prescribed for several indications, including many types of neuropathic pain.

The α _2_δ subunits have a well-established canonical role to influence the trafficking and function of the Ca _V_1 and Ca _V_2 channels, increasing the density of these channels on the plasma membrane ^5^. They also direct trafficking of the channels to specific subcellular sites, including neuronal processes ^5, 6^. In addition, the α _2_δ subunits increase Ca _V_ function by influencing the biophysical properties of the calcium currents ^7– 10^, over and above their effect on trafficking ^6^.

More recently, α _2_δ-1 proteins have been proposed to have non-classic functions of two types: (a) additional functions related to calcium channels, either to link the calcium channel complexes to other proteins or to influence calcium channel function, and (b) roles not associated with calcium channel function.

**Figure 2.**
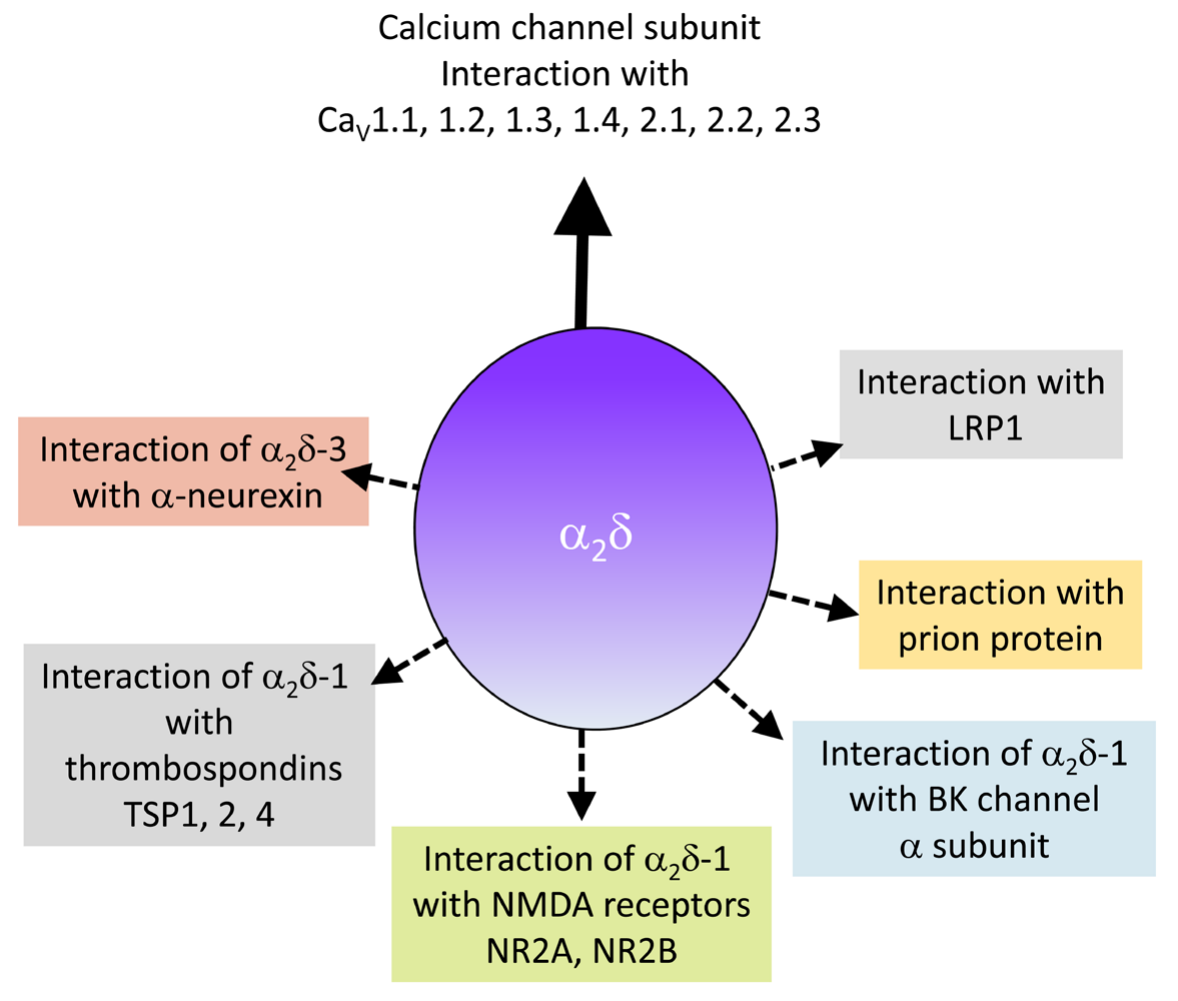
Summary of α _2_δ interactions with other proteins. The various ion channels and other proteins with which α _2_δ subunits have been found to interact are shown. BK, large conductance (big) potassium; LRP1, low-density lipoprotein receptor-related protein 1; NMDA, *N*-methyl- d-aspartate; TSP, thrombospondin.

For (a), I will discuss several topics, including the association of α _2_δ proteins with α-neurexins to influence synaptic transmission ^11, 12^. The α _2_δ-1 protein has also been found to interact potentially with large conductance (big) potassium (BK) channels ^13^, a process which it has been suggested influences calcium channel function by sequestering the α _2_δ subunits. For (b), I will discuss novel roles associated with the association of α _2_δ with thrombospondins (TSPs), an interaction which has been found to influence synaptogenesis in some systems ^14^. I will also discuss the proposed association of α _2_δ with *N*-methyl- d-aspartate (NMDA) receptors ^15^ ( Figure 2). It is possible that the gabapentinoid drugs also act by influencing these various novel targets.

## Topology, domain structure, and biochemical properties of α _2_δ proteins

The α _2_δ subunit was first identified as two proteins—α _2_ and δ—co-purifying as integral constituents of the calcium channel complex present in skeletal muscle T-tubules ^16– 18^. It was found that α _2_δ is encoded by a single gene and is subsequently processed into α _2_ and δ ^17, 18^. Four mammalian α _2_δ genes have been cloned ( *CACNA2D1–4*) ^16, 19– 21^.

**Figure 3.**
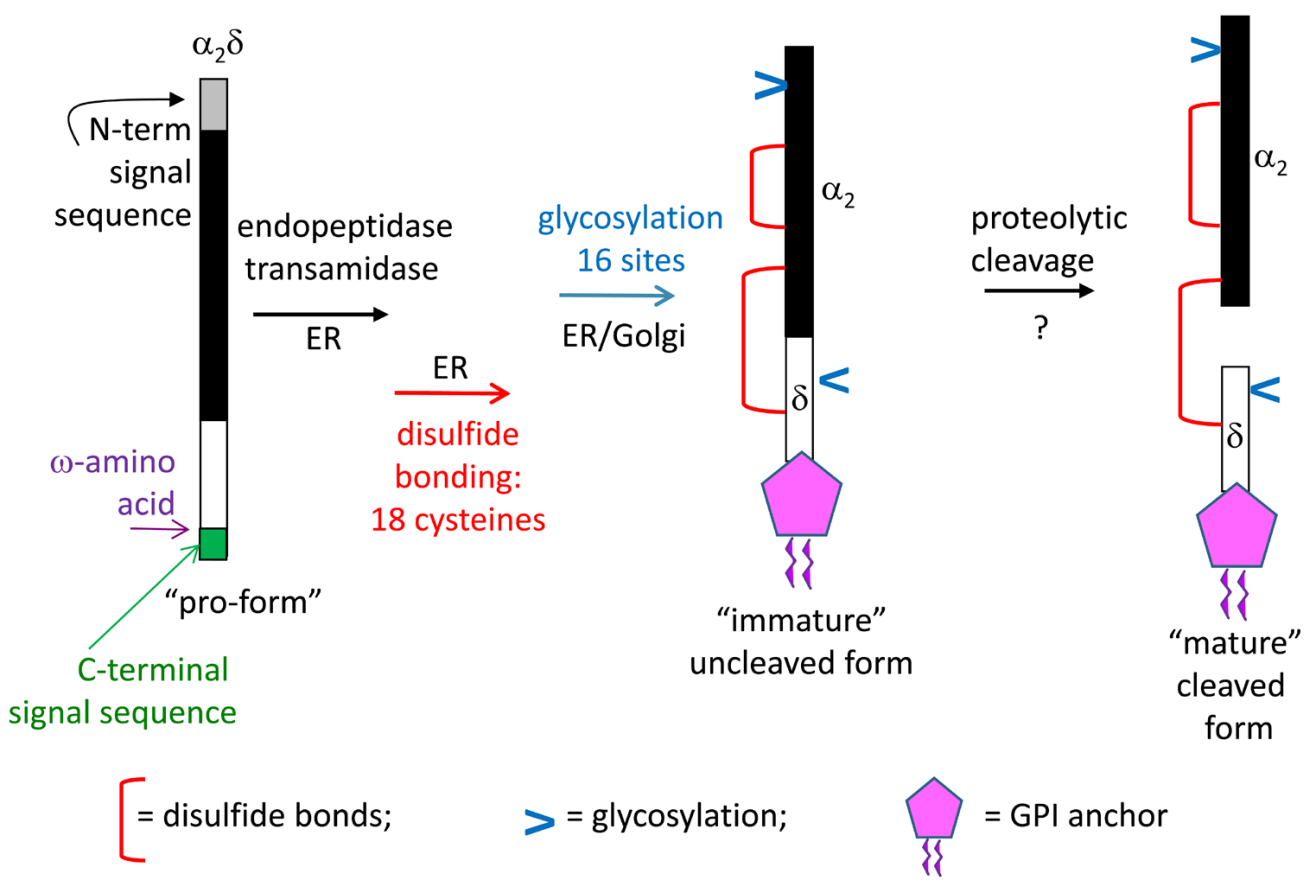
The post-translational processing of α _2_δ subunits. The hydrophobic N-terminal signal sequence is a signal for the polypeptide to co-translationally pass through the membrane of the endoplasmic reticulum (ER). This signal sequence is cleaved off. The glycosylphosphatidylinositol (GPI) anchor is added in the ER by an endopeptidase transamidase, which cleaves the C-terminal signal peptide at the ω-site and adds a pre-formed GPI lipid anchor. Multiple disulfide bonds are formed as the protein folds in the ER, and N-glycosylation occurs at multiple sites. Mature glycosylation is then completed in the Golgi complex, and it is likely that proteolytic cleavage of α _2_δ also occurs here 27. The GPI anchor can also be modified during trafficking.

All the α _2_δ proteins have highly related topology ^22, 23^, with an N-terminal signal sequence, indicating that the N-terminus is extracellular ( Figure 3). The hydrophobic C-terminus of α _2_δ, and its behavior as an integral membrane protein, led to its being categorized as a transmembrane protein ^17, 18^. However, it was subsequently identified to have a strongly predicted glycosylphosphatidylinositol (GPI)-anchor ω-site ^24^. Indeed, multiple pieces of experimental evidence indicate that α _2_δ-1, α _2_δ-2, and α _2_δ-3 (and probably α _2_δ-4 by prediction) are GPI-anchored ^24– 26^.

The α _2_δ subunit genes encode a single precursor protein, which is post-translationally proteolytically processed into two polypeptides. The folding of α _2_δ in the endoplasmic reticulum involves the formation of multiple disulfide bonds both within and between the α _2_ and δ moieties, so that, despite their cleavage, the α _2_ and δ polypeptides remain disulfide-bonded together ^17, 18^. The role for the proteolytic cleavage between α _2_ and δ has been shown to be key to the mature function of these proteins ^6, 28^, and Ca _V_2.2 associates to a greater extent with the mature cleaved form of α _2_δ-1 than with the uncleaved form ^28^.

A von Willebrand factor A (VWA) domain is present in the α _2_ moiety of all α _2_δ proteins ^29, 30^; these widespread domains are generally involved in extracellular protein–protein interactions. A key motif in VWA domains is the metal ion-dependent adhesion site (MIDAS), which involves coordination of the divalent cation by a ring of up to five polar or charged residues ^29^. α _2_δ-1 and α _2_δ-2 have a “perfect” MIDAS site ^30^, whereas α _2_δ-3 and α _2_δ-4 have a missing polar residue ^29^. The α _2_δ subunits also contain multiple Cache domains ^22, 31, 32^, which have homology to domains found in bacterial chemotaxis receptors.

A recent cryo-electron microscopic structural study of the skeletal muscle calcium channel complex provided detailed information on the structure of α _2_δ-1, confirmed the topology of α _2_δ subunits, and identified the interaction sites between α _2_δ and Ca _V_1.1 ^32^, reinforcing the importance of the VWA domain interaction, previously identified ^30^, and also providing evidence for C-terminal GPI anchoring rather than a transmembrane segment associated with α _2_δ-1. The study also identified four sites of disulfide bonding between α _2_ and δ, one of which was found previously by mutagenesis ^33^.

The complex biochemistry of α _2_δ proteins represents a challenge for their study, and it is important to be aware of their distinct biochemical characteristics in terms of their multiple glycosylation sites and disulfide bonds, proteolytic cleavage into α _2_ and δ, and GPI anchoring ( Figure 3). All of these properties might be inadvertently disrupted by the placement of epitope tags or production of mutants, to the detriment of their function ^6, 24, 26, 33^. Furthermore, as elegantly shown very recently with respect to α _2_δ proteins ^12^, co-immunoprecipitation experiments require multiple controls to be sure of the specificity of any interaction, and additional experiments are needed to determine whether any association is direct. This is particularly true when potential binding partners are co-expressed in transfected cells, where elevated concentrations may result in aberrant interactions being detected.

## Properties of α _2_δ as a voltage-gated calcium channel subunit

For the Ca _V_1 and Ca _V_2 channels, α _2_δ universally augments expressed calcium current density ^7– 9, 30^. The α _2_δ subunits also have effects on both kinetic and voltage-dependent properties of the channels, including activation and inactivation. In general, there is a negative shift in the voltage dependence of steady-state inactivation ^30, 34^. In some cases, there is also a hyperpolarization of the voltage dependence of activation, particularly for Ca _V_1.2. Here, it has been shown that α _2_δ-1 mediates a negative shift in voltage-sensor movement in response to depolarization ^35^. There is also an increase in activation and inactivation kinetics ^36, 37^, although these effects depend on the particular α1, β, and α _2_δ subunit used (for a recent review, see 10). Results from co-expression studies (which inevitably lack many components of the native environment) are reinforced by parallel experiments in more intact systems, including using tissues from α _2_δ knockout mice ^20, 38– 42^ and small interfering RNA (siRNA) knockdown of α _2_δ-1 in skeletal muscle cells ^43^ or cardiac myocytes ^44^.

### Role for α _2_δ-1 in calcium channel trafficking

The effect of α _2_δ subunits to increase calcium current density can be partially explained by an increase in the trafficking of the channels to augment the amount on the cell surface ^5^. The exact mechanism whereby α _2_δ increases the density of Ca _V_ channels in the plasma membrane is still unclear. There was no effect of α _2_δ-1 to reduce the internalization of Ca _V_2.2 ^5^, indicating that the effect is likely to be on forward trafficking. Furthermore, the trafficking of α _2_δ itself is blocked by a dominant-negative rab11 construct, suggesting the involvement of the recycling endosomes ^45^.

The VWA domain within the α _2_ moiety of α _2_δ is important for both trafficking of α _2_δ and its associated effect on Ca _V_ channel trafficking and function ^5, 30, 46, 47^. Furthermore, the presence of alternatively spliced exon 37a in the proximal C-terminus of Ca _V_2.2, which is a minor splice variant expressed particularly in certain DRG neurons ^48, 49^, increases Ca _V_2.2 currents ^48^ and also increases its cell surface density via binding to adaptor proteins ^50^. We found that this increase was lost in the absence of α _2_δ subunits, suggesting that this auxiliary subunit promotes particular steps in the forward trafficking process ^50^.

### Proteomic study of Ca _V_2 calcium channels

A comprehensive study of the Ca _V_2 channel proteome was performed by using antibodies against Ca _V_2.1 or Ca _V_2.2, together with antibodies against β subunits, and cataloguing the associated proteins ^51^. Many proteins were found to be part of this complex, although such studies do not indicate whether the interaction is direct or indirect. In contrast to initial purification studies of N-type channels ^52^, and rather surprisingly to many in the field, the interaction of the channels with α _2_δ proteins was found to be much less than 1:1; indeed, it depended on the mildness of the detergent used to solubilize the membranes, resulting in more or less α _2_δ associated with the complex. Since we found that α _2_δ subunits are present in lipid raft fractions ^53^ and subsequently identified that they are GPI-anchored ^24^, this supports the possibility that there is a rather mobile interaction between the α1 and α _2_δ subunits ^53, 54^ or that this interaction is more labile to disruption. Certainly, it also points to a pool of α _2_δ which is not associated with calcium channels, which has also been identified by studies of calcium channel membrane mobility ^54^.

### Importance of studies in knockout mouse models for elucidating potential novel roles for α _2_δ subunits

The genetic ablation of particular α _2_δ subunits has been found to affect neuronal and synaptic morphology in several systems ^56– 58^, pointing to roles for α _2_δ that may or may not involve calcium channels ^22, 59^. Knockout mice have been generated for α _2_δ-1 ^38^, α _2_δ-2 ^20^, α _2_δ-3 ^60^, and α _2_δ-4 ^41^. These have led to important findings regarding both calcium channel function in specific tissues and potential roles for the α _2_δ proteins in neuronal and synaptic morphology and in physiological functions, especially in tissues such as cochlear hair cells ^42^, spiral ganglion neurons ^57^, retinal photoreceptor cells ^58^, and Purkinje neurons ^20, 56^, where one subtype of α _2_δ predominates. However, complementary approaches are also required to elucidate the mechanisms of such effects.

### Importance of α _2_δ in disease states

***Neuropathic pain.*** *Cacna2d1*, encoding α _2_δ-1, is one of many genes whose expression is altered in experimental animals as a result of damage to sensory nerves, which may lead to chronic neuropathic pain. There is a consistent elevation of α _2_δ-1 mRNA and protein ^61– 66^ in every damaged DRG neuron ^39, 62^. Furthermore, we have shown that, in α _2_δ-1 knockout mice ^38^, there is a marked reduction in baseline responses to mechanical and cold stimulation, and a very retarded hyperalgesic response to sciatic nerve injury, in comparison with wild-type littermate mice ^39^.

***Other diseases.*** *CACNA2D1* mutations in humans have been identified to cause cardiac dysfunction, including short QT syndrome ^67^ and Brugada syndrome ^68^. *Cacna2d1* knockout also resulted in a cardiovascular phenotype in mice involving a reduction in basal ventricular cardiac contractility and lower calcium current in ventricular myocytes ^38^. *CACNA2D2* mutations in both humans and mice result in a recessive phenotype including epilepsy and ataxia ^20, 56, 69– 73^, as well as a hearing deficit, related to aberrant trans-synaptic channel organization ^42^. Furthermore, developmentally associated upregulation of α _2_δ-2 expression suppressed axon regeneration in adult spinal cord, although the mechanism remains unclear ^74^. *Cacna2d3* knockout mice have a hearing deficit ^57^ and a central pain phenotype ^60, 75^. Finally, *CACNA2D4* mutations in both humans and mice are associated with night blindness ^76, 77^ and retinal degeneration ^58^.

### Mechanism of action of gabapentinoid drugs which bind to α _2_δ-1 and α _2_δ-2

The α _2_δ subunits are the target for gabapentinoid drugs ^78^, which bind to both α _2_δ-1 and α _2_δ-2 with similar affinity ^79^. However, from studies of mice with mutations in the gabapentin binding site within either α _2_δ-1 or α _2_δ-2, it was concluded that their therapeutic target both in alleviation of neuropathic pain and in epilepsy is α _2_δ-1 ^4, 80^. We have found, from *in vitro* experiments, that incubation with gabapentin lowers the amount of α _2_δ-1 and α _2_δ-2 on the cell surface ^5, 45, 81^ by inhibiting their rab11-dependent recycling to the cell surface ^45^. *In vivo*, chronic administration of pregabalin to sensory nerve-injured rats reduced the elevation in the dorsal horn of pre-synaptic α _2_δ-1, interpreted as being due to inhibition of trafficking ^62^. Thus, gabapentin is likely to influence the function of the other proteins to which these α _2_δ proteins have now been found to bind.

For the relevant Ca _V_ channels, we have also extensively examined the effects of gabapentin. They were initially found to have only small effects on calcium currents when applied acutely ^82^. We found that longer-term incubation of cultured cells with gabapentin produced a clear reduction of calcium currents, both in transfected cells, when α _2_δ-1 or α _2_δ-2 was co-expressed, and in DRG neurons ^45, 81, 83^. We also observed a corresponding reduction in the expression of Ca _V_2 α1 subunits on the cell surface ^5, 45^.

## Other interaction partners for α _2_δ proteins related to their function as calcium channel subunits

Several studies in recent years have provided evidence for novel interactions of proteins with α _2_δ subunits; such interactions then impinge on the function of the calcium channel complex. These interactions may be involved positively in the trafficking of α _2_δ proteins (for example, low-density lipoprotein [LDL] receptor-related protein 1, LRP1) ^27^. By contrast, in several studies, the binding partners have been found to sequester α _2_δ proteins, limiting their access to the Ca _V_ channels, thus reducing both the function and the plasma membrane localization of calcium channels. This mechanism has been proposed for α-neurexins ^11^ and for BK channels ^13^ as well as pathologically for a mutant form of prion protein (PrP) ^84^. These will all be considered in turn.

### Trafficking of α _2_δ-1 by the multifunctional transport protein LRP1

The LRP family represents a large group of ligand-binding and trafficking proteins, including the LDL receptor and LRP1–6. They are multifunctional, multi-domain receptors, interacting with many protein ligands via their ligand-binding domains, mediating both forward trafficking and endocytosis of these ligands ^85^. They are also involved as co-receptors, affecting intracellular cell signaling processes ^86, 87^.

**Figure 4.**
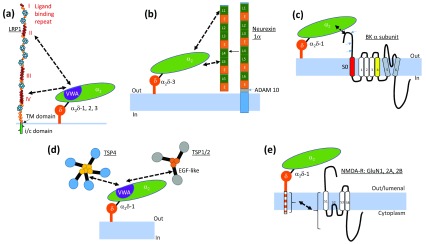
Protein domains involved in novel α _2_δ interactions. ( **a**) Interaction of α _2_δ-1 (and α _2_δ-2/3) with the ligand-binding repeats II and IV of low-density lipoprotein receptor-related protein 1 (LRP1) (red). Other domains in LRP1 are epithelial growth factor (EGF)-like repeats (orange) and β-propeller domains (cyan) ^27^. i/c, intracellular; TM, transmembrane. ( **b**) Interaction of neurexin 1α with α _2_δ-3, via its laminin-like globular (LG) repeats (L, green) 1 and 5. E, EGF-like repeat (orange). Neurexin 1α is cleaved by a disintegrin and metalloprotease 10 (ADAM 10) (arrow) to have the observed effects on synaptic transmission, but it is not clear whether this is required for the interaction with α _2_δ-3 ^11^. ( **c**) Interaction of the extracellular N-terminus of large conductance (big) potassium (BK) α subunits with α _2_δ-1. The three blue arrows indicate the three alternative N-terminal translation initiation sites, the third being the most commonly used ^13^. S0 is the additional transmembrane domain (red). ( **d**) Interaction of α _2_δ-1 von Willebrand factor A (VWA) domain with the EGF-like domains (black bars) of both pentameric (left) and trimeric (right) thrombospondins (TSPs) ^14^. ( **e**) Interaction of a C-terminal region of α _2_δ-1 beyond its GPI-anchor site (dashed orange/white region) with the *N*-methyl- d-aspartate (NMDA) receptor GluN1, GluN2A, and GluN2B subunits ^15^.

LRP1 is a ubiquitous membrane protein with four ligand-binding domains ( Figure 4a) and is involved in forward trafficking of proteins, including several TSPs ^88– 92^, PrP ^93^, and NMDA receptors ^94^. LRP1 is also involved in clathrin-dependent endocytosis ^85, 95^. It is present in synapses ^94^ and is implicated in neurite outgrowth ^96^. Whether different LRP proteins bind to overlapping sets of protein ligands is unclear, but LRP5/6 are also involved in Wnt signaling ^87^.

We recently showed that LRP1 binds to α _2_δ-1 ^27^ and the same is true for α _2_δ-2 and α _2_δ-3 (Ivan Kadurin and Annette Dolphin, preliminary results). For α _2_δ-1, we showed this interaction is direct, involving the VWA domain of α _2_δ-1 and LRP1 ligand-binding domains II and IV ( Figure 4a) ^27^. The association is modulated by the LRP chaperone, receptor-associated protein (RAP), which is required for the correct folding of all LRP proteins and for their trafficking out of the endoplasmic reticulum ^97, 98^. We found that the LRP1/RAP combination increases mature glycosylation, proteolytic processing, and cell-surface expression of α _2_δ-1 and also increases plasma membrane expression and function of Ca _V_2.2 when co-expressed with α _2_δ-1 ^27^. Since LRP1 is able to bind more than one ligand at different sites ^99^, it is possible that it forms a bridge between α _2_δ-1 and other proteins, such as TSPs.

### Sequestration of α _2_δ-3 by interaction with α-neurexins

There are three vertebrate neurexin genes, and each can form α- and β-neurexins from different promoters. The α-neurexins have been found to be important for coupling calcium channels to synaptic transmission ^100^. Whereas in mammalian synapses the neurexins are pre-synaptic and bind to post-synaptic neuroligins, in *Caenorhabditis elegans* this polarity is reversed at many synapses. It has been found in the worm that post-synaptic neurexin 1α at the neuromuscular junction binds, via its laminin-like globular 1 (LG1) domain, to pre-synaptic unc-36 (similar to α _2_δ-3), thus decreasing its availability to bind to the pre-synaptic unc-2 (a Ca _V_2-like channel) that mediates neurotransmitter release ^11^. This was found to reduce synaptic transmission, an effect which required a proteolytically cleaved fragment of neurexin, shed from the post-synaptic plasma membrane ( Figure 4b). In transfected cells, mouse neurexin 1α was found to bind α _2_δ-3 and to decrease Ca _V_2.2 current, whereas there was no effect on Ca _V_2.2 currents in the presence of α _2_δ-1 or α _2_δ-2 ^11^. An attractive suggestion is that this type of pre- to post-synaptic interaction may contribute to trans-synaptic nanoscale organization ^101^. However, in view of recent results described below, it will be important in the future to identify the site of selective interaction on the α _2_δ-3 protein of the LG1 domain (and LG5 in the mouse) ^11^ of neurexin 1α.

In contrast, a more recent article has identified positive effects of neurexin 1α in the presence of α _2_δ-1 (but not α _2_δ-3) on pre-synaptic Ca ^2+^ transients in hippocampal neurons and in parallel on Ca _V_2.1 calcium currents ^12^. Importantly, very carefully done experiments, designed to detect an interaction of neurexin 1α with α _2_δ-1 or α _2_δ-3, failed to find a specific association between the two proteins, as every protein tested (α-neurexin, neuroligin, and two forms of cadherin) was pulled down with α _2_δ-1 (and also α _2_δ-3 co-immunoprecipitated with neurexin 1α). The authors concluded that neurexin 1α does not form stable complexes with α _2_δ subunits but nevertheless influences their function. Their results also provide a warning that α _2_δ proteins may be rather prone to co-immunoprecipitation artefacts.

### Sequestration of α _2_δ-1 by interaction with BK channels

A recent study has identified that BK α subunits bind to α _2_δ-1 subunits via the BK N-terminus ^13^, and the authors suggest that this interaction sequesters α _2_δ-1 from Ca _V_ channels. BK channels are important mediators of cell excitability, as they respond to both voltage and intracellular Ca ^2+^ (for recent reviews, see 102, 103). They consist of a tetrameric pore-forming α subunit, which is unusual compared with other voltage-gated K channels in that it has an additional transmembrane domain (S0), such that the N-terminus is extracellular. Furthermore, the N-terminus of BK α subunits contains an unusual sequence with three translation initiation methionines (M1, 25, and 66 in the human sequence below):

**M ^1^**A N ^3^GGGGGGGSSGGGGGGGGSSLR **M ^25^**SSNIHANHLSLDASSSSSSSSSSSSSSSSSSSSSSVHEPK **M ^66^**DALIIPVTMEVPCDSRGQRM ^86^ WWAFLASSMVTFFGGLFIILLWRTLKYLWTVCCHCGGKTK….

The third start methionine (M ^66^DAL) has generally been thought to be the main translation initiation site ^104^, and the underlined sequence was identified as a novel transmembrane segment S0. There is very good evidence that the existence of this additional transmembrane domain results in an extracellular N-terminus ^104^, although the exact mechanism driving this is unknown, as no signal peptide has been identified. In native rat brain, some mass spectrometry–mass spectrometry (MS-MS) peptide coverage of BK α was also seen from both the first (M ^1^ANG) ^105^ and the second (M ^25^SSN) ^106^ start methionines, indicating that they can also be used. BK channels are modulated by transmembrane β subunits which differentially interact with the different N-terminal isoforms of the BK α subunit and strongly affect BK voltage-dependent properties ^107– 109^. BK channels also interact with γ subunits ^110^.

In the study by Zhang *et al*. ^13^, α _2_δ-1 was found to associate with BK α subunits via their N-terminus ( Figure 4c). This association was found to compete with both Ca _V_1 and Ca _V_2 channels for α _2_δ-1 and therefore reduce the Ca _V_ channel function. Interestingly, the region of BK channels identified by pull-down experiments to interact with α _2_δ-1 is within the N-terminal residues 1–86, which contain two unusual repetitive polyglycine and polyserine stretches (see above). If the sequence encoded from the first start methionine (residues 1–24) was truncated or if the asparagine (N) at position 3 was mutated to D, no effect of the BK channel on Ca _V_α1/β/α _2_δ-1 currents was observed, whereas the *in vitro* binding also involved residues 66–86 ^13^. These results suggest that the effect of BK channels on Ca _V_ channel function would occur only for the full-length BK isoform, starting with MANG. It is also of interest that N3 in the BK channel potentially undergoes rapid deamidation *in vivo* which would abolish its interaction with α _2_δ-1 in a time-dependent manner ^13^, meaning that only a small subset of BK channels might be involved in this interaction with α _2_δ-1. Moreover, in this study, no BK β or γ subunits were expressed and therefore it would be important to determine whether their interaction with the N-terminus or elsewhere would compete with α _2_δ for interaction, which would represent an interesting means of reciprocal cross-talk between these channels.

Because the authors examine the potential role for this BK–α _2_δ-1 interaction for neuropathic pain, in which α _2_δ-1 is upregulated, it would also be of great interest to identify the relative expression from the different translation initiation sites used for the BK α protein in DRG neurons in control and neuropathic states. Furthermore, it should be noted that, in contrast to α _2_δ-1 which is upregulated, BK channel mRNA is downregulated in DRGs following neuropathic nerve injury ^111^.

Surprisingly, in proteomic studies of native rat brain BK channels, α _2_δ was not identified as co-purifying with these channels, although several Ca _V_ channel α1 subunits were well represented ^106^. Ca _V_1.2, Ca _V_2.1, and Ca _V_2.2 as well as the Ca _V_β subunits β1b, β2, and β3 were all found in this study ^106^. Indeed, Ca _V_2.1 was the most abundantly represented protein that co-purified with BK channels, suggesting the possibility of a direct interaction. This finding would seem to contradict the model of Zhang *et al*. ^13^, in which BK competes for α _2_δ with the Ca _V_α1 subunit.

### Sequestration of α _2_δ-1 by interaction with a disease-associated mutant PrP

In an intriguing study, PrP was found to interact with α _2_δ-1 proteins, and a Creutzfeldt–Jakob disease-causing mutant form of PrP resulted in intracellular retention of α _2_δ-1 and disrupted synaptic transmission ^84^. It is of relevance in this regard that both PrP and α _2_δ-1 are GPI-anchored and therefore would be likely to be in similar membrane domains. One confounding issue is that in overexpression studies, α _2_δ-1 and PrP interfere with each other’s trafficking, at least partly because of competition for the limiting supply of GPI anchor ^25^. In this study ^25^, PrP disrupted the ability of α _2_δ-1 to increase calcium currents, but a C-terminally truncated GPI-anchorless PrP did not ^25^. Thus, it remains unclear to what extent the α _2_δ-1 interaction with cellular PrP has a physiological or pathophysiological role ^112^.

## Other interaction partners for α _2_δ proteins, unrelated to calcium channel function

In several studies, new roles independent of calcium channels have been proposed for specific α _2_δ proteins (for example, interaction with TSPs ^14^ and as a subunit of NMDA receptors ^15^). These will now be considered here.

### α _2_δ-1 as a mediator of synaptogenesis via binding to TSPs

TSPs are extracellular matrix proteins which bind to a very large number of proteins, 83 being so far identified for TSP-1 ^113^; consequently, they have many functions ^114– 116^. In the brain, they are produced by astrocytes and promote neurite outgrowth ^117^, including the formation of silent excitatory synapses, lacking post-synaptic receptors ^118^. It was then hypothesized that post-synaptic α _2_δ-1 could be the sought-after post-synaptic binding partner of TSPs to mediate synaptogenesis, independent of any effects on calcium channels. This was first tested using co-immunoprecipitation to determine whether TSPs or individual domains of TSPs interacted with C-terminally tagged α _2_δ-1 ^14^. An interaction which involved a key synaptogenic epithelial growth factor (EGF)-like domain was found ( Figure 4d). As a note of caution, C-terminal tagging may interfere with trafficking of α _2_δ-1 by disrupting the GPI anchor ^24, 26^. Nevertheless, gabapentin was found to inhibit the interaction between α _2_δ-1 and the EGF-like domain of TSP-2 and to disrupt synaptogenesis. Furthermore, *in vivo*, gabapentin was found to disrupt whisker barrel plasticity following whisker removal in some of the mice examined ^14^.

TSP-4 is upregulated in rodent models of neuropathic pain ^119^. Since α _2_δ-1 is also upregulated in DRGs following peripheral sensory nerve injury, several studies have investigated whether an interaction between these two proteins is important in neuropathic pain or the effect of gabapentin. Interestingly, in a recent article, it was suggested that pre-synaptic, rather than post-synaptic, α _2_δ-1 may be a synaptogenic binding partner for TSP-4 in the spinal cord ^120^.

We found (using overexpressed proteins) that TSP-4 modestly reduced the affinity for ^3^H-gabapentin binding to α _2_δ-1, although the effect on ^3^H-gabapentin binding was not reproduced with the TSP-4 synaptogenic EGF-like domain. Furthermore, we found only very weak and unreliable co-immunoprecipitation of the two proteins, which again could not be reproduced with the synaptogenic EGF-like domain of TSP-4 ^121^. We also could not demonstrate any interaction between α _2_δ-1 and TSP-4 on the cell surface of transfected cells, suggesting that the association between these two proteins to disrupt 3H-gabapentin binding is occurring intracellularly following co-transfection, when the two proteins are juxtaposed at high concentration ^121^.

Nevertheless, there is evidence from other studies that α _2_δ subunits are important for synaptic morphology in several different systems ^57, 58, 122, 123^. Whether the role for α _2_δ in calcium channel localization and function is responsible for these morphological changes has not always been investigated. However, α _2_δ was shown to increase pre-synaptic localization of the relevant α1 subunit in Drosophila neuromuscular junction synapses ^124^ as well as in retinal ^58^ and hippocampal ^6^ synapses.

### α _2_δ-1 as an NMDA receptor trafficking protein

It was recently shown that overexpression of α _2_δ-1 administered intrathecally into the spinal cord potentiates pre-synaptic and post-synaptic NMDA receptor activity, and it was further shown that α _2_δ-1 interacted with NMDA receptors, both in spinal cord and in overexpression studies ^15^. The interaction was apparently specific for α _2_δ-1, as it did not occur with α _2_δ-2 or α _2_δ-3. The authors identified the site of interaction as the C-terminus of α _2_δ-1, surprisingly after the C-terminal GPI-anchor cleavage site ( Figure 4e). This was determined using chimeras assembled from the different isoforms, swapping isoforms either between α2 and δ or with the C-terminus of δ ^6^. However, it is important to note that such chimeras may have disrupted the primary sequences involved in proteolytic cleavage between α _2_ and δ, a process which is important for function ^6, 28^, or it might have affected the sequences involved in GPI anchoring ^24^. Nevertheless, this result suggests either that a transmembrane version of α _2_δ-1 may be interacting with NMDA receptors, initially in the endoplasmic reticulum, or that the NMDA receptor interacts with the C-terminal peptide of α _2_δ-1 that is cleaved off during GPI-anchor attachment ^125^.

The GluN1, GluN2A, and GluN2B subunits of NMDA receptors were found to interact with α _2_δ-1, presumably via the transmembrane or intracellular domains of these subunits, since the identified interaction is with the C-terminus of α _2_δ-1 ^15^. The C-termini of these NMDA receptors are rather different in both sequence and function ^126– 128^, and determining the interaction site will be a key next step. It is of interest that α _2_δ-1 has not been previously detected in proteomic studies of post-synaptic densities ^129^. In contrast, other calcium channel subunits (Ca _V_1.2, Ca _V_2.3, and a β) were identified. Another recent study also did not detect α _2_δ-1 when purifying NMDA receptors from mouse brain ^128^, although α _2_δ-1 is widely expressed in most brain regions ^130, 131^. Therefore, it would be important to determine whether this interaction is for some reason observed only in the spinal cord. One possible reason is that it might be indirect (for example, via a scaffolding protein expressed in the spinal cord, interacting with both α _2_δ-1 and NMDA receptors).

## Conclusions and future directions

The α _2_δ subunits are important auxiliary subunits of the Ca _V_1 and Ca _V_2 voltage-gated calcium channels. They play key roles in trafficking of these channels, both to the plasma membrane and to specific subcellular domains, and they have marked effects on the activation and other biophysical properties of these channels, indicating their importance as subunits of the channel complex rather than purely as chaperones. However, recent evidence suggests that they may bind to other proteins, and one role for such additional interactions could be to sequester particular α _2_δ subunits at specific sites away from the calcium channels in a dynamic manner and thus reduce calcium channel function. Evidence also suggests that α _2_δ proteins may independently influence other channels and also affect other functions of neurons. All of these novel functions will need to be critically explored in the future to evaluate further their physiological, pathological, and pharmacological relevance. Furthermore, the roles for novel α _2_δ-like protein, Cachd1, which enhances both T-type channels ^132^ and N-type channels ^133^ as well as competes with α _2_δ-1 ^133^, will be explored further in the future.

## Abbreviations

BK, large conductance (big) potassium; EGF, epithelial growth factor; GPI, glycosylphosphatidylinositol; LDL, low-density lipoprotein; LG, laminin-like globular; LRP, low-density lipoprotein receptor-related protein; MIDAS, metal ion-dependent adhesion site; NMDA, *N*-methyl- d-aspartate; PrP, prion protein; RAP, receptor-associated protein; TSP, thrombospondin; VWA, von Willebrand factor A.
